# Dendritic Cell Tumor Vaccination via Fc Gamma Receptor Targeting: Lessons Learned from Pre-Clinical and Translational Studies

**DOI:** 10.3390/vaccines9040409

**Published:** 2021-04-20

**Authors:** Enrique Gómez Alcaide, Sinduya Krishnarajah, Fabian Junker

**Affiliations:** 1 Roche Pharma Research and Early Development, Pharmaceutical Sciences, Roche Innovation Center Basel, F. Hoffmann-La Roche Ltd., Grenzacherstrasse 124, 4070 Basel, Switzerland; enrique.gomez_alcaide@roche.com; 2 Institute of Experimental Immunology, University of Zurich, 8057 Zurich, Switzerland; krishnarajah@immunology.uzh.ch

**Keywords:** Fc gamma receptors, dendritic cells, vaccination, immune oncology, recombinant immune complexes

## Abstract

Despite significant recent improvements in the field of immunotherapy, cancer remains a heavy burden on patients and healthcare systems. In recent years, immunotherapies have led to remarkable strides in treating certain cancers. However, despite the success of checkpoint inhibitors and the advent of cellular therapies, novel strategies need to be explored to (1) improve treatment in patients where these approaches fail and (2) make such treatments widely and financially accessible. Vaccines based on tumor antigens (Ag) have emerged as an innovative strategy with the potential to address these areas. Here, we review the fundamental aspects relevant for the development of cancer vaccines and the critical role of dendritic cells (DCs) in this process. We first offer a general overview of DC biology and routes of Ag presentation eliciting effective T cell-mediated immune responses. We then present new therapeutic avenues specifically targeting Fc gamma receptors (FcγR) as a means to deliver antigen selectively to DCs and its effects on T-cell activation. We present an overview of the mechanistic aspects of FcγR-mediated DC targeting, as well as potential tumor vaccination strategies based on preclinical and translational studies. In particular, we highlight recent developments in the field of recombinant immune complex-like large molecules and their potential for DC-mediated tumor vaccination in the clinic. These findings go beyond cancer research and may be of relevance for other disease areas that could benefit from FcγR-targeted antigen delivery, such as autoimmunity and infectious diseases.

## 1. Introduction

### 1.1. Cancer Therapy and the Immune System

Cancer remains one of the biggest burdens on healthcare systems worldwide. It is the second major cause of death after cardiovascular disorders. With almost 20 million new diagnoses in 2020 alone and increasing incidences yearly [1], it is clear that the clinical treatment and management of cancers is a continuous challenge for clinicians and researchers alike.

Tumors form as a result of changes at the cellular, genetic, and epigenetic levels in a subset of cells anywhere in the host, a process named carcinogenesis [2]. Aberrant growth cycles and apoptosis evasion allows these precancerous cells to multiply in an uncontrolled fashion. Uncontrolled growth helps these cells to establish a vascularized niche within a tissue, establishing a primary tumor. However, the majority of cancer-related deaths are caused by metastases, a process by which cells from the primary tumor can detach, extravasate into the systemic circulation, and establish themselves in a new tissue. Depending on the type of cancer, the first line of treatment traditionally involves surgery, radiation therapy, or chemotherapy. Primary treatments can be combined with adjuvant treatments to achieve the elimination of the remaining cancer cells with variable success. Importantly, a critical component contributing to tumor growth and maintenance is the fact that cancers may escape effective surveillance by the immune system (IS), for instance, by creating immunosuppressive tumor microenvironmental conditions. Effective tumor immune control is also hampered by the occurrence of “exhausted”, nonfunctional T cells in tumors [3]. Based on these observations, cancer immunotherapy has emerged as a treatment option to harness components of the patient’s IS to fight tumor cells. In cancer immunotherapy, adoptive T-cell transfer therapies, monoclonal therapeutic antibodies (mAbs), and vaccines have been explored. In addition, tumor vaccines aim to educate the IS to recognize and eliminate cells that express tumor-associated Ags, which are, by definition, not present, or only at low levels, in healthy cells [4]. However, the development of vaccines has drawbacks, like the identification of the proper tumor Ags or Ag-derived peptides, the combination with safe adjuvants, and, generally, the need to optimize efficient effector T-cell activation strategies [5].

Importantly, an efficient cancer vaccine must fully activate cytotoxic T lymphocytes (CTLs) that recognize and kill cancer cells. For this, the adaptive IS critically depends on the function of Ag-presenting cells (APCs), including monocytes, B cells, and dendritic cells (DCs). DCs are considered the most professional APCs, since they are specialized in and are able to capture, process, and subsequently present extracellular-derived proteins. Distributed in almost all tissues, they act as sentinels of the IS and are the bridge between innate and adaptive immunity [6]. DCs can generally be grouped into three subsets [7,8]: myeloid/conventional DC1 (cDC1), myeloid/conventional DC2 (cDC2), and plasmacytoid DCs (pDC). The three subsets present Ags with varying efficiency to T cells. Importantly, cDC1 DCs are thought to possess an intrinsic cross-presentation capability. They can effectively activate CD8^+^ T cells, as well as promote CD4^+^ T-helper type 1 (Th1) cells (3), while cDC2 DCs can be induced to cross-present Ags (see below). Thus, they also contribute to CD8^+^ T-cell activation [9]. In addition, pDCs have also been studied in the context of antigen presentation, where they were described to induce antigen cross-presentation post-activation [10,11,12,13,14,15].

### 1.2. DCs Are Crucial for Effective Helper and Cytotoxic T-cell Activation

As suggested before, the effective activation of a T cell fully depends on its interaction with APCs [16]. They require an Ag to be presented in a rather short peptide sequence in a peptide:protein complex by the APC. The full activation of T cells requires the interplay of three different signals. Signal 1 is the recognition of the specific peptide presented to the T-cell receptor (TCR) by the major histocompatibility complex (MHC) molecules (either MHC-I for cytotoxic CD8^+^ T cells or MHC-II for CD4^+^ T cells) presented on APCs [17,18]. Restricted MHC-I peptides are mostly of cytoplasmic origin, while restricted MHC-II peptides are of extracellular origin [18]. This process itself is not sufficient to trigger the effective activation of Ag-specific T cells. In addition, they require Signal 2, characterized by the interaction between costimulatory molecules on T cells (e.g., CD28) and their counterparts on the APCs, such as CD80 and CD86 (also termed B7.1 and B7.2). Finally, to define the type of response, Signal 3 is required in the form of cytokines. Together, these three signals induce Ag-specific CD4^+^ or CD8^+^ T-cell responses [19]. Since DCs have the special ability to ingest virus-infected cells or tumor cells, they are able to present Ags derived from these to specific CD8^+^ T cells. The DCs activate them through a process termed cross-presentation *via* a separate MHC-I pathway [9,20,21].

**Figure 1:**
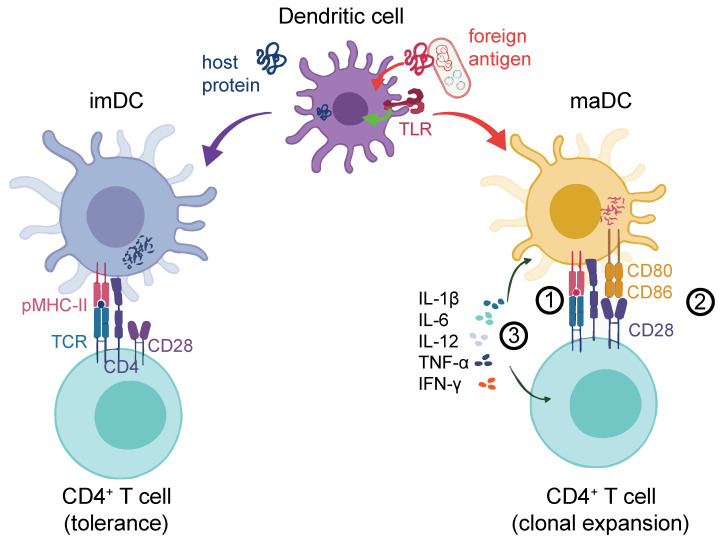
DC response to the antigen challenge. DCs can process either host-derived (self) proteins (blue, left-hand side) or foreign antigens (red, right-hand side). The latter could be from an exogeneous source (e.g., bacteria as illustrated) or cancer cell-derived neo-Ags. Self-protein processing and the presentation to T-cell receptors (signal 1) *via* peptide–MHC complexes (pMHC) leads to tolerance. In addition to signal 1, foreign antigens can lead to a strong DC activation, for instance, through the co-stimulation of TLRs or other receptors (not shown), which entails the upregulation of co-stimulatory molecules such as CD80 or CD86 at the DC surface (signal 2). These prolong and intensify the TCR-driven activation of antigen-specific T cells. Finally, cytokines such as IL-1β, IL-12, IL-6, IFN-γ, and TNF-α are released (signal 3) by both the DC and the T cell, which further shape the antigen-induced T-cell response. TCR: T cell receptor; pMHC-II: Peptide-MHC-II receptor; imDC: Immature DC; maDC: Mature DC.

DCs themselves become activated upon contact with foreign Ags [22]. DC activation can occur upon the engagement of conserved bacterial or viral Ags, so-called pathogen-associated molecular patterns (PAMPs) *via* pattern recognition receptors (PRRs). In resting conditions, immature DCs (imDCs) are equipped with several types of PRRs, including Toll-like receptors (TLRs), membrane-associated C-type lectin receptors (CLRs) [23], and mannose receptors [24,25,26,27,28,29]. Following the recognition of pathogens, imDCs can remain in a tolerogenic state [30] or undergo a maturation process where they lose their endocytic ability while increasing the Ag processing and presentation capacity [31,32]. PRR engagement activates mitogen-activated protein kinase (MAPK) and nuclear factor kappa-light-chain-enhancer of activated B cell (NF-κB) signaling [33], which, in mature DCs (maDCs), induces the expression of proinflammatory cytokines such as tumor necrosis factor alpha (TNF-α), interleukin-12 (IL-12), and IL-6 [34]. This is particularly important for the activation and clonal expansion of proinflammatory Th1-type CD4^+^ T cells [35]. MaDCs also upregulate chemokine receptors like CCR7 that drive their homing to lymph nodes (LN) [36,37]. The secretion of cytokines is reflected in a profound transcriptional change in DC gene expression that also results in the upregulation of Signal-2 markers, such as MHC-II, CD80, CD86, and CD40 [38,39]. Importantly, DC activation may alternatively trigger anti-inflammatory Th2 CD4^+^ T-cell activation or invigorate other specialized T-helper subsets, such as Th17, Th22, or regulatory T cells (Treg), depending on the context. We illustrate an overview of proinflammatory DC-mediated T-cell activation [40] in Figure 1.

PRRs are also relevant with regards to vaccine development, where the effective activation of T cells is critical. Although from a mechanistic point of view, targeting DCs seems like a promising avenue for vaccine development; it has been demonstrated that many DC vaccines alone do not achieve full T-cell activation [41]. In an effort to overcome this limitation, adjuvants can be used as key molecules aimed to promote stronger T-cell responses by inducing DC maturation and prolonging their exposure to antigens [41]. The efforts to create effective adjuvants have focused on the use of microbial compounds and selective TLR ligands [42,43,44]. However, PRRs are not only expressed on APCs but on a wide variety of myeloid cells, including neutrophils [45], monocytes [46,47], and macrophages [46], as well as nonimmune cells such as endothelial cells [48]. Consequently, the use of microbial compounds seemed to elicit a very broad inflammatory immune cell activation caused by non-DC PRR activation. Currently, even though adjuvants like TLR ligands [26,49,50], aluminum, or saponin-based particles are being studied to maximize the immunogenicity of vaccines [51,52,53,54], this strategy may still entail the risk of inducing general inflammation.

### 1.3. FcγR Crosslinking on DCs Leads to Effective T-Cell Activation and Proliferation

In addition to PRRs, DCs express Fc-gamma receptors (FcγR) that can lead to a highly effective internalization of Ag and subsequent DC activation [55]. FcγRs, when crosslinked through IgG antibody-complexed (“opsonized”) soluble Ag, allow for rapid internalization and cellular activation [56,57]. This immune complex (IC) will then be shuttled to endolysosomal DC compartments, where the Ag will subsequently be degraded. This facilitates the subsequent MHC:peptide generation and Ag presentation on the DC [56,58]. In humans, three groups of FcγRs have been described across a variety of cell types: FcγRI (CD64), FcγRIIA/B (CD32A/B), and IIIA/B (CD16A/B) [59]. Concerning DC activation, all FcγRs except FcγRIIB are considered activators; FcγRIIB acts as an inhibitory receptor. FcγRs bind—albeit, with different affinities [60]—to the Fc (fragment, crystallizable) portion of IgG antibodies [58]. On DCs, the expression of FcγRs depends on the cell subtype. FcγRI is expressed on monocyte-derived DCs (moDCs) [61]. FcγRIIA has been described on conventional DCs, which were also shown to express FcγRIIB [62]. In addition, human, as well as murine, pDCs were described to express FcγRII [63,64,65,66]. Overall, human and mouse DCs express largely overlapping FcγR subsets [67,68]. However, the balance between activating and inhibitory receptors on APCs critically depends on the tissue of origin, and the numbers of cell surface receptors can be different between the species [69].

Several lines of evidence, both in humans and mice, have convincingly demonstrated that IgG:Ag ICs induce a superior DC activation compared to the delivery of uncoated, “naked” Ag [56,57]. For example, pulsing in vitro human moDCs with polyclonal tetanus IgG ICs led to a stronger increase in DC activation, including the release of proinflammatory cytokines compared to “naked” tetanus toxoid Ag [57,70]. Similarly, in a pivotal mouse study, ovalbumin (OVA) preincubated with anti-OVA IgG was taken up much more efficiently by splenic mouse DCs ex vivo than “naked” OVA. Importantly, in mice transplanted with OVA-specific CD8^+^ or CD4^+^ T cells, OVA:IgG ICs induced both CD4^+^ and CD8^+^ T-cell proliferation more efficiently than “naked” OVA [71]. Similar experiments were repeated with henn egg lysozyme IgG ICs and with mice selectively lacking activating, as well as inactivating, FcγRs [55,71,72]. In addition, mechanistic confirmation was achieved using mouse models where signal transduction downstream of FcγRI and FcγRIIIA was impaired [55,73]. Another study showed that IC-mediated FcγR crosslinking in mouse DCs was required to induce long-lasting transcriptional changes reflected in the induction of T cell-polarizing genes, such as *IL2*, *IL6*, *IL10*, *IL15*, *IL23a*, *IL27*, and *Ifnb1* [74]. These experiments provide the mechanistic basis to target FcγRs *via* IgG ICs, and this holds promise for DC-specific vaccination strategies [75].

## 2. Targeting DCs for Cancer Vaccination *via* FcγRs: Mechanistic Principles

### 2.1. Allogenic Tumor IgG ICs Can Trigger Cancer Immunity via DC Activation

Tumor rejection is thought to rely largely, if not completely, on the host’s effective immune response to tumor cells [76,77]. This process entails the immunosurveillance of potentially tumorigenic host cells by an intricate interplay between APCs and effector cells [78]. In cancer patients, DCs can present tissue-associated Ags or neo-Ags, which originate through cancer-specific DNA alterations [78,79,80]. This has led to the development of DC-selective tumor vaccination strategies [75,81,82].

In principle, both humoral, as well as cytotoxic T cell-mediated host immune responses, can lead to tumor rejection, depending on the tumor immunogenicity [83,84]. Interestingly, in mouse tumor systems such as the C57/BL6 B16F10 melanoma model [85,86,87], the rejection of tumors in allogenic recipient animals can be observed, suggesting pre-existing allogenic tumor immunity where immunocompetent mice reject allogenic (but not syngeneic) tumor cells post-transplantation. In a pivotal mouse study, Carmi et al. systematically assessed the mechanistic basis of allogenic tumor immunity and found that it was initiated by naturally occurring tumor-binding IgG, which enabled DCs to internalize tumor Ags and, subsequently, activate tumor-reactive T cells. Allogeneic tumors contained more maDCs than syngeneic tumors. The authors found that IgG binding to tumor cells was critical to initiate DC activation by using allogenic IgG fractions in conjunction with tumor cell lysates, thus generating tumor Ag:IgG ICs. Importantly, tumor Ag presentation following an antibody-mediated uptake by DCs was sufficient to initiate protective T cell-mediated immunity. This was confirmed in human cancer, where healthy donor IgG could form ICs with allogenic patient-derived lung carcinoma lysates. These and other results [71,88,89,90] prompted more mechanistic analyses of Ag:IgG IC-mediated cancer immunity.

### 2.2. FcγR-Targeted Vaccination Strategies in Preclinical Tumor Models

Mouse ex vivo cancer vaccination protocols involving the DC Ag challenge were developed as early as the 1990s [91,92]. As outlined before, the IgG IC:FcγR axis may provide an even more attractive angle for the design of DC-targeted strategies [93,94], leading to the development of IgG IC-mediated tumor vaccination models.

In an early congenic mouse melanoma model [95], the OVA-expressing B16F10 cell line MO-4 was used. Bone marrow-derived DCs (BMDCs) were generated from wildtype (wt) C57/BL6 animals and challenged in vitro with rabbit IgG:OVA ICs or “naked” OVA. IC:BMDC recipient animals were almost completely protected from tumor engraftment, while all control animals developed melanomas. This vaccination was much more efficient in inducing T-cell responses and longer-lasting compared to BMDCs challenged with “naked” OVA. In a recall experiment, OVA mice that had been vaccinated with the IgG IC BMDC protocol and subsequently survived MO-4 tumor cell transplantation were re-challenged with MO-4 a half-year later, and none of the animals developed palpable tumors. More importantly, from a therapeutic point of view, 40% of the tumor-bearing mice transplanted with OVA:IgG IC-challenged BMDCs could be rescued. This suggested that targeting DCs with IgG ICs might be exploited in tumor prevention, as well as in tumor treatment. Lastly, using C57Bl.6 β2M^−/−^, transporter associated with antigen-processing 1 (TAP1)^−/−^, MHC-II^−/−^ and FcγRγ^−/−^ animals, the authors confirmed that the vaccination depended on FcγRs and induced both MHC-I- and MHC-II-restricted responses.

Another mouse study by Schuurhuis et al. using OVA-expressing B16 tumor cells (here: MO-5) confirmed that the in vitro BMDC challenge with Ag:IgG IC s was superior to “naked” Ag stimulation [96]. Here, the differential contribution of mouse FcRγs was assessed by the selective and/or combined knockout (KO) of specific FcγRs. These comparisons showed that FcγRI and FcγRIII were required for enhancing the cross-presentation of CD8^+^ T cells, the critical effector T cells. *In vitro*, as well as in vivo, assays showed that FcγRI was found to compensate for the absence of FcγRIII and vice versa. Consequently, in this model, activating (but not inhibitory) FcγRs on BMDCs were required for the efficient priming of Ag-specific CD8^+^ T cells and induction of tumor protection. This confirmed again that, in tumor vaccination protocols, MHC-I^−/−^ or MHC-II^−/−^ DCs are unable to induce T cell-mediated tumor protection downstream of the DC Ag:IgG IC challenge [95]. Importantly, further experiments confirmed that transplanting BMDCs matured in vitro was more protective compared to mere OVA:IgG IC administration in a MO-5 melanoma induction model.

The Ag:IgG IC vaccination also induces functional humoral antibody responses to tumor Ags. In a recent study by Kim et al. [97], a recombinant Ag antibody IC was generated. Through the production of recombinant GA733, an epithelial cell adhesion molecule (EpCAM), Fc fusion protein, and an anti-GA733 mAb, anti-GA733 IgG ICs were obtained. These were subsequently administered to immunocompetent mice, leading to the induction of a Th2 response followed by the generation of (presumably polyclonal) anti-GA733 mouse antibodies. This antiserum was found to delay the growth of a human EpCAM^+^ colorectal cancer cell line in a nude mouse model. Upon tumor manifestation, serum derived from mock-challenged, GA733-challenged, or antibody:GA733 IC-challenged immunocompetent mice were transfused into recipients. Here, the antibody:GA733 IC-derived serum was found to be significantly superior at controlling tumor growth compared to GA733-challenged serum vaccination.

Taken together, IgG:Ag ICs provide an attractive entry route for therapeutic anticancer DC vaccination protocols with clear advantages over vaccinations with “naked” Ag. Importantly, the use of the whole Ag protein over human leukocyte antigen (HLA)-restricted peptide-based [98] tumor vaccination protocols could also mean more patients would be eligible for such treatments.

## 3. The Long Way to the Clinic: Lessons Learned from Translational Models

### 3.1. Ag:IgG IC or Ag plus Hapten?

Haptens are small molecules engineered in such a way that, in combination with a larger carrier such as a protein [99,100,101], they can elicit the production of antibodies that bind specifically to it. Haptens were first described by Karl Landsteiner, who demonstrated that molecules with a molecular weight lower than 1 KDa cannot elicit an immune response [102]. Only when a hapten–protein complex is formed can it be recognized by a DC and, therefore, lead to an immune response. Consequently, for the effective use in the form of a therapy, these molecules need to be covalently bound to a protein in a reaction termed haptenization. Some studies suggest that DC-targeted vaccination strategies employing hapten:tumor Ags, or hapten:whole-tumor cell preparations combined with the use of anti-hapten immunization to boost the DC response led to vaccination success [103,104]. On the flipside, this entails the risk of unwanted cytokine release and nonspecific inflammation. In addition, hapten-based strategies are challenging from a practical point of view, since the technical procedure might be quite complex. Specifically, patient tumor cells may need to be cultured, followed by patient vaccinations and a subsequent re-challenge with multiple immunogenic stimuli (i.e., hapten challenge followed by the transplantation of hapten-coated tumor cells). Through their ability to target Ags to APCs whilst simultaneously activating them, IgG:Ag ICs could represent an elegant strategy to avoid the use of haptens in tumor vaccination.

IgG ICs can be highly variable [105], and a disadvantage of full-length Ag:IgG ICs [97] is their undefined size and valency, a factor to keep in mind especially in the case of polyclonal ICs. From a clinical point of view, this intrinsic heterogeneity and potential folding alterations may hamper the establishment of clear molecular modes of action (MoA), a requirement for rational drug design. This is also critical with regards to restricting the Ag:IgG IC uptake to DC subsets with high intrinsic Ag presentation potential while minimizing uncontrolled inflammation and potential allergic reactions [82]. These observations, together with the different IgG idiotype affinities exerted by the various FcγRs, entail different opportunities for the development of recombinant therapeutic IgG Fc-based IC designs, which mostly focus on the generation of Fc-based multimeric constructs.

Another important component of an immune response is the complement system, an innate response consisting of a network of over 50 different proteins [106]. Whilst the role of the complement system in many pathologies—such as allergies—has been widely studied and defined as specific subtypes of hypersensitivity reactions (immune complex-mediated or type III reactions) [107], its role in cancer is still elusive. There are several studies suggesting that a complement may play a role in modulating immunosuppression within the tumor microenvironment, as reviewed elsewhere [108]. In particular, c5a can potentiate Ag processing and presentation by human DCs [109]. Therefore, a c5a-based vaccination therapy has been tried in a murine model of melanoma [110]. Whilst the focus of this review is the cellular component of the immune response, the role of complement in tumor vaccination was summarized by Reis et al. [106].

### 3.2. Recombinant IgG ICs to Target Human FcγRs

Most of the more advanced recombinant therapeutic IC candidates have a valency between three and six IgG Fc domains linked to each other. In a pivotal study, Ortiz and colleagues designed multimeric Fc polymers with valences ranging from *n* = 2 to *n* = 5 [111] by fusing the human IgG1 Fc domain with the human IgG2 hinge sequence [112]. They found that a low Fc valency (*n* = 3) led to high avidity binding to FcγRs but did not lead to cellular activation, whereas higher valency constructs (*n* = 5) led to effective FcγR activation. This was assessed by immune tyrosine-activating motifs (ITAM) signaling downstream of IC binding. In contrast, the trivalent construct Fc3Y did not induce cellular activation. Instead, it inhibited FcγR-mediated responses to disease-associated ICs isolated from systemic lupus erythematosus (SLE) patient sera in a variety of human immune cells. The flow cytometry assessment suggested that Fc3Y was bound to FcγRIIA^+^ and FcγRIIIB^+^ granulocytes, FcγRIIIA^+^ natural killer (NK) cells, and FcγRI^+^ FcγRIIA^+^ FcγRIIB^+^ FcγRIIIA^+/-^ monocytes. Critically, it bound to DCs, which predominantly expressed FcγRI, FcγRIIA, and FcγRIIB. There was minimal binding to B cells, suggesting preferential binding to activating FcγRs. Conclusively, the authors suggested that the further development of Fc3Y could be used in autoimmune diseases to dampen APC activation cascades. This could eventually replace the current therapeutic gold standard, high-dose intravenous immunoglobulin (IVIg) (45). However, these findings also suggest the principal possibility of using recombinant IgG ICs to target DCs for vaccination.

More recently, Spirig et al. pursued a similar strategy by using hexameric recombinant IgG1-Fc fusion proteins [113]. The authors generated their IC-like molecule (termed Fc-μTP-L309C) by fusion of the IgM μ-tailpiece to the C-terminus of human IgG1 Fc. They found that Fc-μTP-L309C inhibited FcγR-mediated effector functions, such as antibody-dependent cell-mediated cytotoxicity (ADCC) or phagocytosis in vitro. In addition, it suppressed inflammatory arthritis in mice when given therapeutically at much lower doses than IVIg in a comparable fashion to Fc3Y [111]. The finding that a higher-valency recombinant Fc fusion protein effectively led to the in vitro and in vivo downregulation of FcγR signaling is, at first glance, a contradiction of the findings by Ortiz et al., which determined that only low-valency constructs avoid ITAM-mediated cell activation. Interestingly, Spirig et al. claimed to observe an increased calcium influx in Fc-μTP-L309C-challenged monocytes pinpointing to FcγR crosslinking-driven cell activation. However, they claim that this stimulus is not sufficient to induce proinflammatory cytokine release.

Another study by Mekhaiel et al. also used the hexameric IgG1-Fc fusion protein as a recombinant IC [114]. These cylindrical molecules confirmed the binding to human and mouse FcγRs on immune cells, including human B cells. This is the first study using recombinant, hexameric IgG1 Fc-based ICs for vaccination purposes. In a mouse model of malaria where mice were challenged with infected erythrocytes of *Plasmodium berghei* transgenic for the Merozoite surface protein 1, C-terminal 19-kDa region (MSP1_19_), the authors assessed the BALB/c in vivo generation of *Plasmodium falciparum* MSP1_19_-specific IgG1 Ab titers post-challenge with multimeric IgG1:MSP1_19_ constructs. Dimeric and hexameric complexes were administered subcutaneously or intraperitoneally. BALB/c mice transgenic for human FcγRI were also used. Murine anti-MSP1_19_ antibodies were produced only if MSP1_19_ was administered in an IC form. Interestingly, the hexameric complex was found to be less efficient as a vaccine. The authors claimed that the protective effect was not sufficient, suggesting ineffective immunological memory generation. The route of administration had no effect on the outcome, and human FcγRI transgenic animals were not better protected than their wt littermates. Speculatively, in this mouse setting, the rather disappointing vaccination results might be explained by a preferential engagement of murine FcγRs expressed on granulocytes; Mekhaiel et al. consequently suggest exploring other, non-IgG1-based Fc fusion constructs for a more effective vaccination.

Finally, another set of hexameric human IgG Fc-based IC-like molecules were designed to target FcγRs in autoimmunity. Qureshi and colleagues [70] generated fully human IgG1 Fc or IgG4 Fc-derived constructs. In a similar fashion to Spirig et al. [113], they multimerized through the insertion of the IgM tailpiece. In order to improve the protein yield and minimize phagocytosis, as well as platelet and complement activation, different Fc multimer versions were designed. Due to the relatively unaltered protein sequences used, a minimal risk for immunogenicity was expected. FcγR engagement by these molecules was mostly avidity-driven. Importantly, in vitro, in a macrophage-labeling experiment, Fc multimers were efficiently internalized and shuttled to recycling endosomes. This strongly suggests that, through IgG IC-like constructs, Ag cargo would be delivered to the APC compartments where the processing of extracellular-derived Ags occurs, a prerequisite for MHC-mediated peptide presentation and T-cell activation [56]. Critically, a significant degradation of the stimulatory FcγRs was observed after contact with hexameric Fc, while FcγRIIB was not affected. The authors then assessed the functional consequences of this on phagocytes and APCs. Interestingly, while macrophage-driven phagocytosis was inhibited post-FcγR engagement, Ag presentation was not. Here, in a polyclonal tetanus:IgG IC model, tetanus-induced T-cell proliferation was not significantly reduced at high doses of hexameric Fc. In this model, DCs are assumed to be the major T cell-activating cell type [57]. Given the purported MoA of Fc multimers resulting in the degradation of activating FcγRs, this would advocate for a critical role of FcγRIIB in IC-mediated T-cell activation, at least in the case of poorly defined polyclonal tetanus:IgG ICs.

When administered to mice or *cynomolgus* monkeys, hexameric Fc was cleared from the serum rapidly, something to consider for potential clinical applications, requiring challenging dosing regimens in humans. Importantly, the authors observed the transient elevation of interleukin-6 (IL-6) and IL-10 in mice but were unable to detect interferon-γ (IFN-γ), tumor necrosis factor alpha (TNF-α), or IL-1β, suggesting only transient FcγR-mediated cellular activation. However, in a follow-up study using the same array of engineered molecules in human in vitro safety assays, Rowley et al. found that IgG1 Fc hexamers triggered a proinflammatory cytokine release in a whole-blood assay [115]. Neutrophils were found to be main drivers of IFN-γ and TNF-α release post the Fc multimer challenge. In contrast, a stimulation with IgG4 Fc hexamers did not induce cytokine release in this assay. These results were mimicked in another safety assay, where the platelet activation post-Fc multimer challenge was assessed. Here, the IgG1 Fc hexamer was found to induce platelet activation between 15% and 60%, whilst the IgG4 Fc hexamer was not strongly activated in platelets. Finally, using statistically designed mutagenesis, the authors suggested that L234 and K274 might be critical for FcγRIIA-mediated platelet activation. They also found this residue to be critical in Fc hexamer-mediated IFN-γ release. It also led to even more decreased phagocytosis capacity, presumably by altering the IgG Fc affinity to distinct FcγRs.

Finally, Kim et al. designed hexameric antigen:IgG Fc ICs, which they termed polymeric immunoglobulin G scaffolds (PIGS). Similar to other hexameric IgG Fc multimers [70,113], multimerization was also achieved using a C-terminal IgM-derived μ-tail piece [116]. This construct was assessed in the context of viral infection. Concretely, mouse IgG2a and human IgG1 versions were created where the consensus domain III sequence (cEDIII) of dengue glycoprotein E was linked to the IgG Fc CH domains CH2 and CH3 by a short peptide sequence. In mice, after subcutaneous administration, cEDIII-PIGS induced dengue-specific IgG responses that could be boosted by an aluminum hydroxide gel (alum) co-challenge, while cEDIII alone was fully ineffective without the alum. Even with alum, it induced a weaker antibody response compared to cEDIII-PIGS + alum. This also resulted in superior dengue virus serotype 2 neutralization. In addition to inducing humoral responses, it induced IFN-γ, IL-2, and IL-17, producing mouse T cells in a splenocyte cEDIII recall experiment. In a follow-up study using the human IgG Fc multimer version, superior T-cell activation and intracellular proinflammatory cytokine production in CD4^+^, as well as CD8^+^ T cells, was also observed in human tonsil cell cultures challenged with cEDIII-PIGS [117]. These experiments further showed that cEDIII-PIGS engaged FcγRI, FcγRIIA, and FcγRIIIA, which is expected for IgG1-Fc derived molecules [60,68]. However, Kim et al. did not assess the possible in vivo safety signals, such as platelet activation or the release of proinflammatory cytokines, in the periphery.

**Figure 2:**
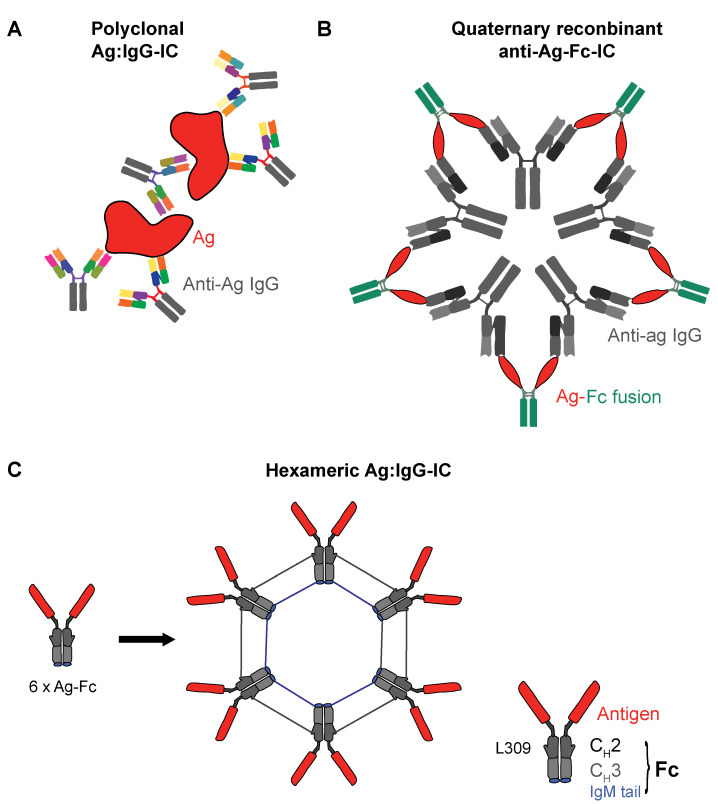
Conceptual overview of Ag:IgG ICs. (**A**) Oligo- or polyclonal Ag:IgG ICs are often used in basic research. They can be of highly variable size and Fc valency. Naturally occurring Ag:IgG ICs may also be made up of different IgG idiotypes. (**B**) Quaternary anti-Ag-Fc complexes are composed of defined anti-Ag mAbs, which aggregate with recombinant Ag-Fc fusion proteins as exemplified by Kim et al. This noncovalent binding leads to effective FcγR crosslinking and potentially highly efficient Ag uptake by DCs, but the concrete structure of the Ag-FC:IgG ICs remains undefined and highly variable. (**C**) Fully recombinant, IgG-Fc-derived Ag fusion proteins can form cylindrical hexameric structures, as exemplified by Mekhaiel et al., or cEDIII-PIGS by Kim et al. The hexameric covalent polymerization of the IgG-Fc-Ag fusion proteins can be achieved by introducing mutations to the CH2 and CH3 domains of the IgG-Fc part (at L309) and/or the addition of the IgM μ-tail piece.

We summarized and illustrated various constitutions of polyclonal and recombinant IgG Fc-based ICs in Figure 2.

### 3.3. How Translatable Are Preclinical IC Vaccination Models?

Whilst in mouse models, the key role of FcγRs in the response induced by therapeutic mAbs has been well-demonstrated, in humans their role has been more elusive, due to the genetic variations or polymorphic differences present among individuals [118]. These affect several features of FcγRs—such as levels of receptor expression, affinity, or activating/inhibitory capacity [119,120]. In addition to this biological variation, the FcγR expression levels on certain PBMC subsets may be reported differently on frozen vs. freshly prepared material, which can be a critical factor in the comparison of datasets from different experiments [118]. Many preclinical DC vaccination tumor models use BMDCs, which, in a clinical setting, are not readily available, unlike patient peripheral blood mononuclear cells (PBMCs). Thus, a potential source for human DCs could be PBMC-derived moDCs [31]. Mouse [121] and human [57] moDCs can be readily differentiated, cultured, and activated in vitro with IgG ICs. However, due to their lengthy differentiation and intensive in vitro manipulation, moDCs were found to achieve only modest clinical response rates in cancer vaccination trials, raising the question if moDCs represent the best candidates for human DC vaccination [122]. In addition to in vitro manipulation-associated changes in DC biology, in vivo biological species singularities may also account for potential differences in translational studies comparing mouse and human DCs [123]. Finally, the immunological status of the animal or patient (naïve vs. inflamed or tumor bearing) [123,124,125], as well as the subject’s age [126,127], may affect the DC subset numbers, tissue distribution, and biology. Particularly important is the number of FcγRs on the surface of mouse and human APCs and the ratio of activating vs. inhibitory receptors. A recent study quantifying FcγRs in mouse and human PBMCs concluded that, for certain FcγRs, substantial species differences needed to be taken into account [69]. Concretely, the number of FcγR subtypes was highly different, which, in turn, affected the ratio of activating vs. inhibitory receptors. After the Ag:IgG IC challenge, this could be crucial to determine if an APC becomes activated or not [128]. Furthermore, on human monocytes, FcγRIIB was extremely variable between different donors, which may reflect differences in the IgG IC-mediated activation potential. In addition, various FcγR variants have been described in humans, with effects on the functionality and IgG idiotype affinity [60]. Even though exhaustive quantitative FcγR flow cytometry expression data are not available for human DC subsets, this highlighted the complexity of FcγR biology on APCs, which may be critical for the understanding of translational cancer vaccine studies. Other species differences are relevant concerning pDCs. For instance, CD1c^+^ moDCs, but not pDCs, were found to be able to prime CD8^+^ T cells and induce MHC-I Ag peptide presentation in humans [129], while the capacity of mouse pDCs to internalize Ag:IgG ICs is still under debate. Some reports claim FcγRIIb expression on pDCs [63] while others claim these findings to be due to cDC contaminants [7].

### 3.4. Advantages and Challenges of Recombinant ICs as DC Targeted Vaccines

Preclinical and translational studies suggest that, when directly co-administered with an antigen, IgG ICs lead to sustained Ag presentation and the induction of long-lasting T-cell memory in vivo. Without additional hapten administration, IgG ICs entailed the *bona fide* licensing of DCs to induce strong tumoricidal CD8^+^ CTL responses [95] when BMDCs where vaccinated ex vivo. A possible explanation for the effectiveness of this protocol is most likely that FcγR crosslinking is fully sufficient to effectively activate DCs so that the “license to kill” [130] requirement no longer applies. This would mean that cytotoxic CD8^+^ T cells with antitumor properties could be primed by IC-challenged BMDCs in the absence of CD4^+^ Th cell signaling. A disadvantage of many preclinical models is the use of poorly defined ICs. However, fully recombinant IgG ICs have now been produced by several groups and hold great potential for vaccination purposes and can, in principle, be fine-tuned to target specific subsets of FcγRs [131]. Besides cancer, IgG ICs also hold great potential for other disease entities, especially due to their intrinsic customization potential [128].

However, for vaccination purposes, a major drawback seems to be that direct Ag:IgG IC administration (without generating BMDCs first) may not lead to long-lasting T-cell memory, as reported using a mouse infection model [114]. This, together with the pharmacokinetics (PK) profile expected for IgG ICs [70], makes direct Ag:IgG IC administration challenging in favor of an ex vivo DC challenge. However, as opposed to BMDCs, moDCs seem to be less suited for ex vivo DC vaccinations, whilst other populations such as cDCs may not be obtainable in large enough quantities from cancer patients. Even though this is occasionally done in a clinical targeted vaccination context [132,133], the suitability of a patient peripheral blood DC isolation and ex vivo challenge protocol, especially for multicenter clinical trials, is debatable. This is particularly true with regards to cellular retention, which remains a critical problem with DC vaccination therapies. For instance, it has been reported that cell counts can drop to around 50% post-cryopreservation [132], which directly affects the therapeutic applicability of such protocols.

Whether the benefits of ex vivo DC manipulation will outweigh the challenges of direct recombinant Ag:IgG IC administration, especially in the context of multicenter trials, remains to be seen. If recombinant IgG ICs are to be administered directly, co-stimulations with CD40L or other DC activators may be necessary as an additional boost [129].

### 3.5. FcγRs in Clinical Trials: More Than a Biomarker?

For the potential use of recombinant Ag:IgG ICs targeting DCs in cancer vaccination, it is important to consider the discrepancies in the results obtained depending on the IC valency, highlighting the intrinsic complexity of FcγR biology in APCs. The fine art of establishing a model that selectively and fully activates DCs without activating other immune cell populations is the key to successfully translating these strategies into clinical applications.

Together with the difficulties to generate the perfect adjuvant, there is extensive literature describing single-nucleotide polymorphisms (SNPs) associated with FcγRs [134] and their functional implications. Concretely, SNP analyses are applied as biomarkers to evaluate the response to therapies involving humanized IgG1 mAbs like elotuzumab (anti-CD319) [135], rituximab (anti-CD20) [136,137,138], obinutuzumab (anti-CD20) [139], or cetuximab (anti-epidermal growth factor receptor) [140]. These mAbs are thought to lead to direct cell killing, which is, at least in part, driven by FcγRIIIA expressing NK cells [141]. However, as described earlier, mAb binding to cancer cells may also lead to phagocytosis and the activation of APCs inducing T-cell priming [86,142]. In summary, in humans, the differential contribution of FcγRIIIA to ADCC [143,144] and FcγRIIA to Ag presentation through Ag:IgG ICs has been robustly demonstrated [145]. Consequently, therapeutic Ag:IgG ICs should be designed to primarily engage FcγRIIA in DCs. However, despite efforts to study the role of FcγRs as modulators of the immune response through vaccines [146], there is no conclusive dataset supporting their use in a clinical setting. Therefore, the value of these receptors as the main target to elicit full DC maturation entailing T-cell responses in human clinical trials remains elusive. This is reflected by the fact that no clinical trials are currently investigating the benefits of IgG ICs targeting FcγRs either as adjuvants or recombinant Ag:IgG IC cancer vaccination treatment.

We believe that some of the major challenges are related to the Fc multimeric structure of these compounds and their concomitant intrinsic tendency to induce proinflammatory cytokines released in the blood. This can be accompanied by elevated platelet activation (especially through IgG1:ICs), thus posing a potential safety risk for the patient. Importantly, these safety concerns could be addressed in the future through specific IgG-Fc amino acid point mutations. This would alter the binding of the IC to selective FcγRs, thus mitigating, for instance, platelet activation through FcγRIIA engagement [147]. Importantly, however, it could also result in reduced DC activation upon direct Fc multimer:Ag administration. It remains unclear if a balance between effective DC activation through (activating) FcγR crosslinking can be achieved while simultaneously reducing the unwanted activation of non-APCs. Importantly, it will equally be critical to design high-avidity Ag:IgG ICs to avoid a relatively recent phenomenon termed ITAM-mediated inhibitory signaling (ITAMi) [148]. ITAMi suggests that the suboptimal crosslinking of activating FcγRs may lead to cellular inhibition, whereby low-avidity FcγR crosslinking results in the recruitment of the Src homology region 2 domain-containing tyrosine phosphatase (SHP-1), eventually triggering anti-inflammatory immune reactions.

**Figure 3:**
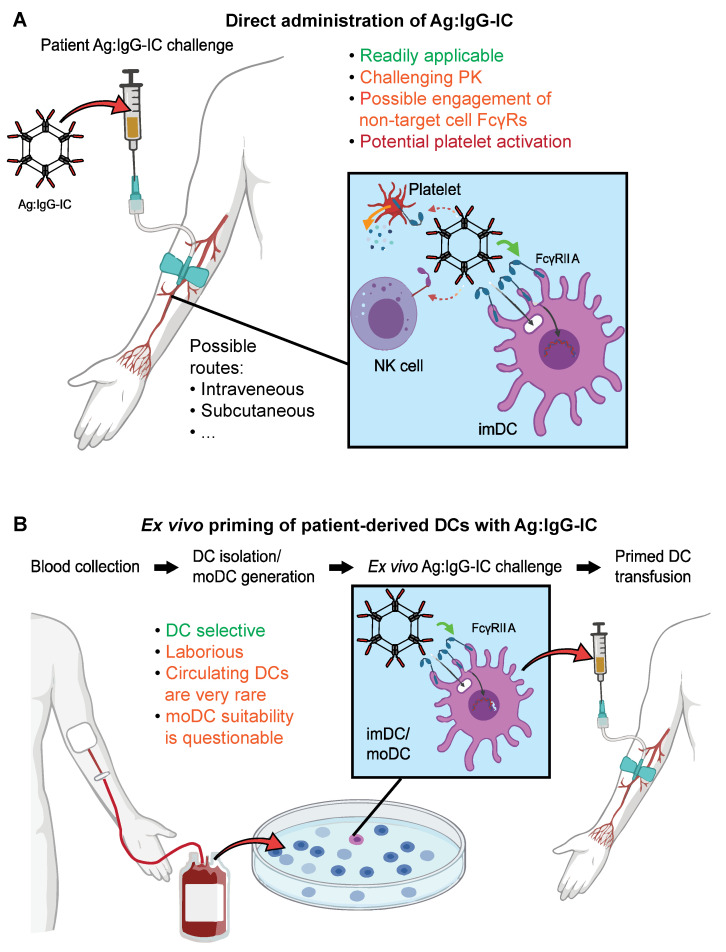
Comparison of the proposed protocols of Ag:IgG IC DC vaccination. (**A**) Direct administration of Ag:IgG ICs to patients. This route is readily applicable and only requires recombinant, Figure 1. which may entail unwanted cytokine release. The expected PK profile is challenging due to its quick clearance. (**B**) Autologous DCs can be primed ex vivo and, subsequently, be administered to the patient *via* adoptive transfer protocols. This route is more laborious and requires readily available patient-derived DCs of high antigen-presenting capabilities. While moDCs can be prepared from patient blood, in vitro differentiation further prolongs the procedure, and the suitability of moDCs for this process is questionable. NK, natural killer cell; moDC, monocyte-derived DC; imDC, immature DC.

In summary, future efforts should be invested in generating Fc multimer constructs that allow to specifically activate DCs independently of FcγRs polymorphisms and avoid the activation of nontarget populations. In principle, two protocols for Ag:IgG IC DC vaccinations are conceivable and have been tried in clinical settings: a direct in vivo challenge with recombinant Ag:IgG IC or ex vivo DC challenge. These are outlined in Figure 3.

## 4. FcγRs as DC Targets for Tumor Vaccination: Concluding Remarks

In the field of cancer immunotherapy, different DC-targeted vaccination protocols have been developed, and some of them have been assessed in clinical trials using a variety of different strategies. These include, among others, DNA vaccines [149] but, also, peptide vaccinations and the use of larger constructs such as synthetic therapeutic peptide conjugate vaccines [150] to induce polyclonal T-cell activation through Ag:IgG ICs. Though conceptually promising, they all come with different limitations.

Activating DCs *via* FcγR crosslinking through recombinant IgG ICs holds great potential for cancer vaccination for several reasons. Firstly, due to the relatively limited expression of FcγRs, the direct administration of IgG ICs may preferably activate APCs as opposed to other leukocytes or nonimmune cells, especially if the Fc component is “fine-tuned” for affinity and selectivity. Secondly, IgG ICs could be used as an adjuvant or directly coupled to a tumor-associated antigen. The latter strategy would open up a potentially large patient population as no prior HLA restriction applies, and a polyclonal T-cell response is to be expected. On the flipside, substantial knowledge gaps remain with respect to the functional effect of FcγR polymorphisms in the patient population. Additionally, the exact administration of IgG ICs is critical, with a preference for the ex vivo stimulation of DCs for the most efficient activation. Importantly, if direct IgG IC administration is considered, safety concerns need to be taken into account already at the design phase of the IC. Finally, due to species differences affecting both the affinity of FcγRs in model systems as well as their expression patterns, there is a clear need for a comprehensive quantification of FcγRs in DCs and other immune cells in the most relevant translational model species: mice, *cynomolgus*, and humans.

## Abbreviations

ADCC	Antibody-dependent Cell-mediated Cytotoxicity
Ag	Antigen
alum	Aluminum hydroxide gel
APC	Antigen-presenting cell
BALB/c	Bagg and Albino mouse strain
BMDC	Bone marrow-derived dendritic cell
CD	Cluster of differentiation
cDC	Conventional dendritic cell
cEDIII	Consensus domain III sequence
CLR	C-type lectin receptors
CTL	Cytotoxic T lymphocyte
DC	Dendritic cell
DNA	Deoxyribonucleic acid
EpCAM	Epithelial cell adhesion molecule
Fc	Fragment, crystallizable
FcγR	Fc-gamma receptors
HLA	Human Leukocyte Antigen
IC	Immune complex
IFN-γ	Interferon-γ
Ig	Immunoglobulin
IL	Interleukin
imDC	Immature dendritic cell
IS	Immune system
ITAM	Immunoreceptor tyrosine-based activation motif
ITAMi	ITAM-mediated inhibitory signaling
IVIg	Intravenous immunoglobulin
KO	Knockout
LN	Lymph node
maDC	Mature dendritic cell
mAb	Monoclonal antibody
MAPK	Mitogen-activated protein kinase
MHC	Major histocompatibility complex
MoA	Mode of action
moDC	Monocyte-derived dendritic cell
MSP1-19	Merozoite surface protein 1, C-terminal 19 kDa region
NF-κB	Nuclear factor kappa-light-chain-enhancer of activated B cells
NK	Natural killer cell
OVA	Ovalbumin
PAMP	Pathogen-associated molecular patterns
PBMC	Peripheral blood mononuclear cell
pDC	Plasmacytoid dendritic cell
PIGS	Polymeric immunoglobulin G scaffold
PK	Pharmacokinetics
PRR	Pattern recognition receptor
SHP-1	Src homology region 2 domain-containing tyrosine phosphatase
SLE	Systemic lupus erythematosus
SNP	Single Nucleotide Polymorphism
TAP1	Transporter associated with Antigen Processing 1
TCR	T cell receptor
Th	Helper T cell
TLR	Toll-like receptors
TNF-α	Tumor necrosis factor alpha
Treg	Regulatory T cell
wt	wildtype
